# Unwanted Hormonal and Metabolic Effects of Postoperative Adjuvant Mitotane Treatment for Adrenocortical Cancer

**DOI:** 10.3390/cancers12092615

**Published:** 2020-09-14

**Authors:** Vittoria Basile, Soraya Puglisi, Anna Calabrese, Anna Pia, Paola Perotti, Alfredo Berruti, Giuseppe Reimondo, Massimo Terzolo

**Affiliations:** 1 Internal Medicine, Department of Clinical and Biological Sciences, San Luigi Gonzaga Hospital, University of Turin, 10043 Orbassano, Turin, Italy; basile_vittoria@libero.it (V.B.); soraya.puglisi@unito.it (S.P.); a.pia@sanluigi.piemonte.it (A.P.); paola.perotti@unito.it (P.P.); giuseppe.reimondo@unito.it (G.R.); massimo.terzolo@unito.it (M.T.); 2 Medical Oncology, Department of Medical and Surgical Specialties, Radiological Sciences, and Public Health, Spedali Civili Hospital, University of Brescia, 25123 Brescia, Italy; alfredo.berruti@gmail.com

**Keywords:** adrenocortical carcinoma, adrenal insufficiency, endocrine effects, hypercholesterolemia, hypogonadism, hypothyroidism, mitotane, ovarian cyst, statin, toxicity

## Abstract

**Simple Summary:**

Mitotane is the only drug approved for treatment of adrenocortical cancer. Although mitotane is a derivative of the pesticide dichlorodiphenyltrichloroethane (DDT), limited data are available on its toxicity. Herein, we reported on the type and frequency of mitotane adverse events and on supportive therapies used to deal with toxicity in 74 mitotane-treated patients. Beyond the expected glucocorticoid insufficiency, a significant number of patients had a deficit of mineralocorticoid hormones, hypothyroid state and impaired testicular function, while fertile women frequently developed ovarian cysts during mitotane treatment. Multiple hormone replacement therapies were needed in >30% of patients. Statins were used in 50% of patients for significant hypercholesterolemia. Supportive therapies were able to revert the biochemical alterations, although higher doses were frequently used due to pharmacokinetic interactions with mitotane. Our study underlines the need of a careful and global approach to manage mitotane toxicity, to make adjuvant therapy safer and easier for patients.

**Abstract:**

Mitotane is widely used for the treatment of adrenocortical cancer (ACC), although the drug-related toxicity complicates its use. The aim of this study is to assess comprehensively the different endocrine and metabolic unwanted effects of the drug, and to provide data on the supportive therapies. We retrospectively analyzed 74 ACC patients adjuvantly treated with mitotane for ≥12 months. During the treatment period (40 months, 12–195), 32.4% of patients needed replacement therapy for mineralocorticoid deficit, 36.2% for hypothyroidism and 34.3% for male hypogonadism. In fertile women, hypogonadism was uncommon, while 65.4% of women developed ovarian cysts. Although no significant change in low-density lipoprotein (LDL) was observed, statins were started in 50% of patients for a significant increase in total cholesterol and triglycerides. Dyslipidemia occurred early, after a median time of 6 months from mitotane start. Conversely, testosterone replacement was usually started after >2 years. In many cases, ranging from 29.4% to 50% according to the side effect, toxicity occurred well before the achievement of the target mitotane concentrations. Supportive therapies were able to revert the biochemical alterations induced by mitotane, although higher doses were needed for a likely pharmacokinetic interaction of exogenous steroids and statins with mitotane. In conclusion, adjuvant mitotane therapy is associated with a spectrum of unwanted effects encompassing the function of different endocrine glands and requires a careful clinical and biochemical assessment associated with the therapeutic drug monitoring.

## 1. Introduction

Medical therapy for adrenocortical carcinoma (ACC) has progressed slowly and is still based on mitotane, a drug introduced in the 1960s for the treatment of inoperable ACC [1]. Mitotane seems well-suited for the scope, as the drug possesses both antihormonal and antitumoral effects [2]. The antihormonal effect is explained by the capability of the drug to inhibit a number of adrenal enzymes involved in steroid synthesis [3], while the basis of the cytotoxic effect remains to be fully elucidated. According to one of the most accepted views, mitotane impairs the activity of sterol-O-acyl transferase 1 (SOAT1), also named acyl-coenzyme A:cholesterol acyltransferase 1 (ACAT1) and leads to the intracellular accumulation of free lipids, thus causing endoplasmic reticulum stress that promotes apoptosis and cell death [4]. This mechanistic hypothesis may explain why mitotane exerts an adrenotoxic effect since sterol-O-acyltransferase-1 (SOAT1) expression is exceedingly high in the adrenal cortex [5,6].

Given that mitotane is a metabolite of the pesticide dichlorodiphenyltrichloroethane (DDT), there is no wonder that its use in humans is fraught with many unwanted effects [2,3,7]. In old series, the use of mitotane was associated with severe neurological adverse events, including lethargy, dizziness and somnolence that were found in 40% to 60% of patients causing significant disturbance of ordinary life activities [8]. Interestingly, SOAT1 is also expressed in the brain and this may explain the tropism of mitotane for the brain [9]. However, it has been later recognized that severe central nervous toxicity is tied to very high mitotane concentrations, thus introducing the value of therapeutic drug monitoring [10,11,12].

After mitotane monitoring became a standard of care in expert centers, reports of severe neurological toxicity induced by mitotane have remarkably abated. In a recent survey, at referral centers for ACC care in Italy, we found that toxicity associated with chronic mitotane treatment in an adjuvant setting was acceptable [13]. The current use of a low-dose monitored mitotane regimen is likely key to avoid overtreatment and consequent toxicity. Despite all the preventive measures that can be put in place to minimize mitotane-related toxicity, it remains true that mitotane has a narrow therapeutic index and can induce a wide spectrum of unwanted effects that render its use complex [2,14]. Management of mitotane treatment is further complicated by the lack of a systematic evaluation of both drug-related toxicity and the outcome of supportive therapy. The limited evidence available has precluded the formulation of clear recommendations for treatment of mitotane-induced adverse effects in the European Society of Endocrinology (ESE)–European Network for the Study of Adrenal Tumors (ENSAT) guidelines for the management of ACC [15].

The aim of the present study is to retrospectively review the experience of our center at San Luigi Gonzaga Hospital with the management of the unwanted effects of adjuvant mitotane treatment given as a low-dose, monitored regimen.

## 2. Results

The baseline characteristics of the 74 patients included in the study are reported in Table 1.

In our series, there were 35 males and 39 females; the median age at diagnosis was 46 years (range 18–77), ACC was at stage II in most cases, secretion was present at diagnosis in 51.4% of patients and cortisol was the most frequently secreted hormone, alone or in combination with other steroid hormones (especially androgens).

**Figure 1:**
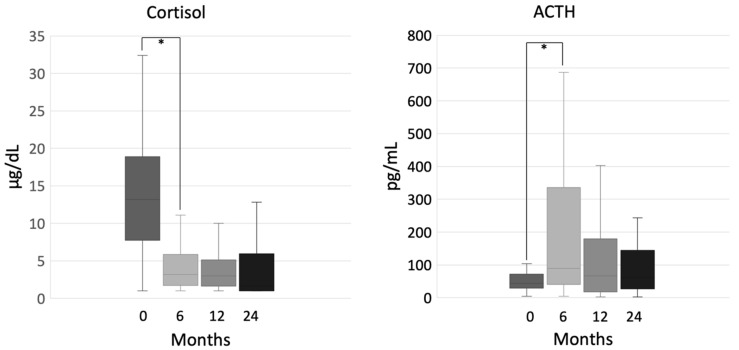
Cortisol and adrenocorticotropic hormone (ACTH) during mitotane therapy; * indicates statistical significance.

In the overall group, 17 patients (23%) interrupted follow up before 24 months—12 of them because of recurrent disease that was treated with systemic therapy. Two patients were lost at follow up before 24 months, and 3 patients had not yet completed the first two years of treatment at the time of study end. After six months of mitotane treatment, cortisol levels were markedly reduced (14.6 μg/dL (1.4–32.4) at baseline vs. 3.2 μg/dL (1–11.1) at 6 months, *p* = 0.016) and adrenocorticotropic hormone (ACTH) levels raised (45 pg/mL (8–104) at baseline vs. 89 pg/mL (5–687) at 6 months, *p* < 0.001), while no statistically significant variation in hormone levels occurred at the following time-points (Figure 1).

**Figure 2:**
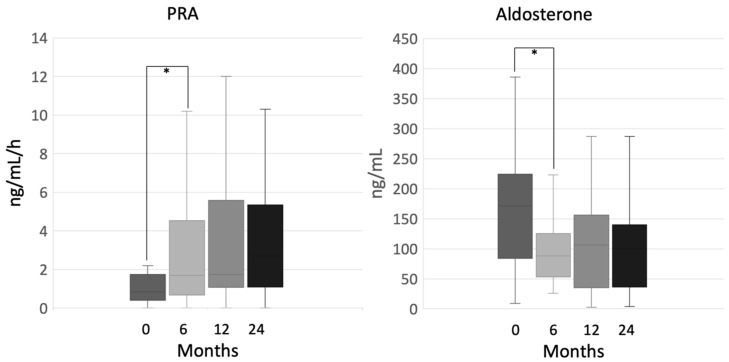
Plasma renin activity (PRA) and aldosterone during mitotane therapy, * indicates statistical significance, Note: patients who were put on mineralocorticoids replacement were excluded from the analysis.

After six months, plasma renin activity (PRA) values were markedly increased (0.9 ng/mL/h (0.2–2.2) at baseline vs. 1.7 ng/mL/h (0.1–10.2) at 6 months, *p* = 0.003), and aldosterone significantly decreased (171 ng/mL (9–386) at baseline vs. 88 ng/mL (26–223) at 6 months, *p* = 0.004), while no statistically significant variation occurred at the following time-points (Figure 2).

Table 2 summarizes the type and the timing of supportive therapies.

All patients received hydrocortisone (or equivalent steroid coverage) at initiation of adjuvant mitotane therapy, with a median hydrocortisone-equivalent dose of 0.67 mg/kg (0.22–1.20). Mineralocorticoid replacement was necessary in 16 patients (21.6%) during the first two years of mitotane therapy, and in 24 patients (32.4%) during the whole period of follow up. After 6 months of fludrocortisone therapy, we observed a nonsignificant reduction in PRA, with no change in aldosterone and potassium levels (PRA, 5 ng/mL/h (0.4–50) at baseline vs. 2.2 ng/mL/h (0.52–23.5) at 6 months, *p* = 0.16; aldosterone, 52.5 ng/mL (23.8–215) at baseline vs. 69.4 ng/mL (27–153) at 6 months, *p* = 0.73; potassium, 4.6 mEq/L (3.2–5.5) at baseline vs. 4.1 mEq/L (3.2–4.7) at 6 months, *p* = 0.07).

**Figure 3:**
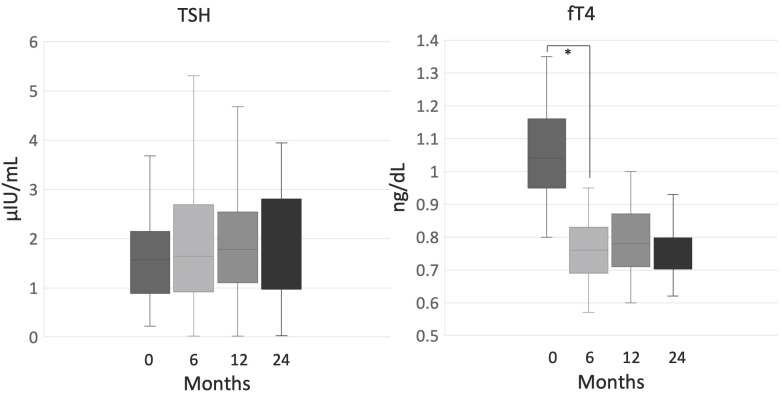
Thyroid-stimulating hormone (TSH) and free thyroxine (fT4) during mitotane therapy; * indicates statistical significance, Note: patients who were put on levothyroxine replacement were excluded from the analysis.

Thyroid-stimulating hormone (TSH) and free thyroxine (fT4) levels were analyzed in 69 patients, since 5 patients were already on levothyroxine for various thyroid disorders. After 6 months, levels of fT4 significantly decreased (fT4, 1.04 ng/dL (0.8–1.35) at baseline vs. 0.76 ng/dL (0.57–0.95), *p* < 0.001), while no statistically significant variation occurred at the following time-points during mitotane treatment. Conversely, TSH did not significantly change either after 6 months (TSH, 1.57 uUI/mL (0.22–3.68) vs. 1.64 uUI/mL (0.02–5.31), *p* = 0.068, or during the whole observation period (Figure 3).

Levothyroxine therapy was started in 19 patients (27.5%) during the first two years, and in 25 patients (36.2%) considering the whole period of follow up. After 6 months of substitutive treatment with levothyroxine, we registered a significant increase in fT4 and a decrease in TSH levels (fT4, 0.67 ng/dL (0.56–0.87) vs. 0.78 ng/dL (0.63–0.99), *p* = 0.002; TSH, 1.74 uUI/mL (0.15–6.52) vs. 0.82 uUI/mL (0.05–4.14), *p* = 0.001).

**Figure 4:**
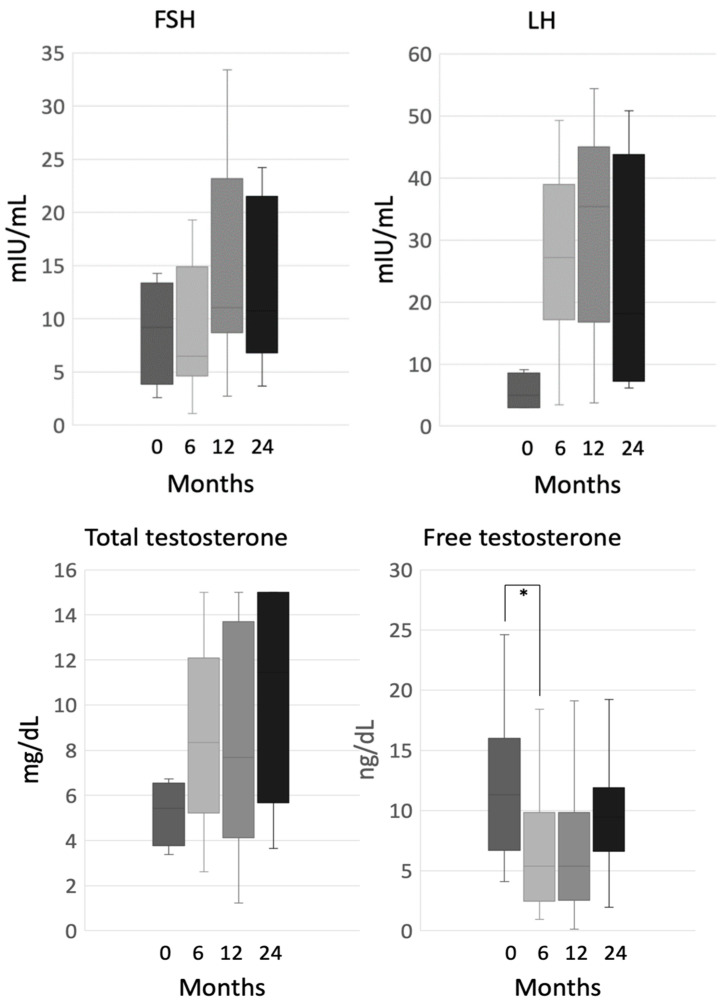
Follicle-stimulating hormone (FSH), luteinizing hormone (LH), total and free testosterone during mitotane therapy in male patients; * indicates statistical significance, Note: patients who were put on testosterone replacement were excluded from the analysis.

We evaluated the pituitary-gonadal axis in 35 male patients, and we observed a nonsignificant increase in follicle-stimulating hormone (FSH), luteinizing hormone (LH) and total testosterone levels, either after 6 months (FSH, 9.2 mIU/mL (2.6–14.3) at baseline vs. 6.5 mIU/mL (1.1–19.3) at 6 months, *p* = 0.18; LH, 5.0 mIU/mL (2.9–9.1) at baseline vs. 27.1 mIU/mL (8.4–49.3) at 6 months, *p* = 0.18; total testosterone, 5.4 mg/dL (3.4–6.7) at baseline vs. 8.3 mg/dL (2.6−15) at 6 months, *p* = 0.26), or during the whole period of observation (Figure 4). Conversely, in the first six months, sex hormone-binding globulin (SHBG) levels significantly increased (45.8 nmol/L (27.2–130) at baseline vs. 180 nmol/L (20.7–180) at 6 months, *p* = 0.003), and free testosterone values significantly dropped (11.3 ng/dL (4–24.6) at baseline vs. 5.3 ng/dL (0.9–18.4) at 6 months, *p* = 0.035), while no statistically significant variation occurred at the following time-points (Figure 4).

Testosterone replacement therapy (testosterone enanthate) was introduced in 5 patients (14.3%) during the first two years of mitotane treatment, and in a total of 12 patients (34.3%) considering the whole follow up. After 6 months of replacement therapy, we observed a significant increase in total and free testosterone levels (total testosterone, 9.6 ng/mL (1.2–12.9) vs. 13.2 ng/mL (0.1–16), *p* = 0.02; free testosterone, 6.6 ng/dL (2.5–13.2) vs. 9.84 ng/dL (0.06–15.6), *p* = 0.01), without significant changes in gonadotropin levels (FSH, 11.8 mIU/mL (4.6–26.6) vs. 13.8 mIU/mL (8.1–39.1), *p* = 0.08; LH, 30.6 mIU/mL (20.4–30.1) vs. 34.2 mIU/mL (22.3–46.4), *p* = 0.18).

We evaluated the lipid panel in 70 patients, while 4 patients were excluded because of pre-existing statin treatment.

**Figure 5:**
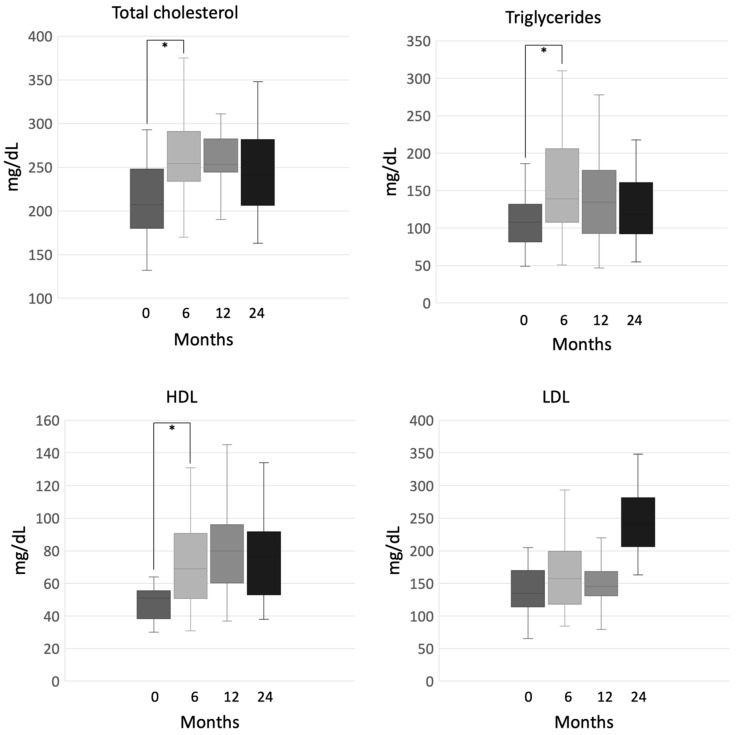
Total cholesterol, triglycerides, high-density lipoprotein (HDL) and low-density lipoprotein (LDL) cholesterol during mitotane therapy, * indicates statistical significance, Note: patients who put on lipid-lowering therapy were excluded from the analysis.

After 6 months, triglycerides, total and high-density lipoprotein (HDL) cholesterol significantly increased (total cholesterol 207 mg/dL (132–293) at baseline vs. 254 mg/dL (170–375) at 6 months, *p* = 0.001; HDL cholesterol 51 mg/dL (30–64) vs. 69 mg/dL (31–131), *p* = 0.004; triglycerides 108 mg/dL (49–186) vs. 139 mg/dL (51–310), *p* = 0.03), while no statistically significant variation occurred at the following time-points during mitotane treatment (Figure 5). Low-density lipoprotein (LDL) cholesterol showed a trend to increase, but we did not observe any significant change either after 6 months (135 mg/dL (65–205) at baseline vs. 157 uUI/mL (85−293) at 6 months, *p* = 0.13), or during the whole observation period (Figure 5).

During the first 2 years of mitotane treatment, lipid-lowering therapy was started in 29 patients (41.4%) and in 35 patients (50%) during the whole follow up period. After 6 months of lipid-lowering therapy, we observed a significant decrease in total cholesterol (305 mg/dL (215–574) vs. 257 mg/dL (181–452), *p* < 0.001) and LDL cholesterol levels (216 mg/dL (111–455) vs. 138 mg/dL (98–357), −33% (−63%–+14%), *p* < 0.001), without any significant change in HDL cholesterol (71 mg/dL (46–134) vs. 90 mg/dL (45–155), *p* = 0.25) and triglycerides (129 mg/dL (12–645) vs. 128 mg/dL (52–258); *p* = 0.05). Rosuvastatin was the most frequently used drug: in 10 patients at the dose of 10 mg/daily (28.6%) and in 15 at the dose of 20 mg/daily (42.8%). In the group of patients treated with rosuvastatin at 10 mg/daily, the LDL cholesterol reduction was −6% (−59%–+14%) (173 mg/dL (120–380) vs. 147 mg/dL (97–299)), while in patients treated with rosuvastatin at 20 mg/daily, the reduction was −44% (−63%–+2%) (217 mg/dL (146–455) vs. 121 mg/dL (103–182)). In 2 patients, we used ezetimibe because of intolerance to statin. The remaining patients assumed pravastatin, fluvastatin or simvastatin.

Among the 39 women in our study, 13 were in postmenopausal state at the time of diagnosis and did not develop any ovarian cysts. Of the 26 fertile women, ovarian cysts were present before mitotane start in 3 patients, while in the remainders, we observed new ovarian cysts (of at least 2 cm) in 17 patients (65.4%)—on average 8 months (2–87) after the introduction of mitotane therapy. In most of these women, no specific treatment was necessary beyond close monitoring with routine imaging (Table 3).

Finally, we evaluated the time relationship between the onset of side effects (evaluated as the time when a specific treatment was instituted) and the achievement of target mitotane levels (≥14 mg/dL). Except for testosterone therapy, many patients were started on supportive therapies regardless of the achievement of target mitotane levels (Table 4).

## 3. Discussion

In the present study, we performed a systematic assessment of the endocrine and metabolic toxicity of mitotane in a rather large cohort of ACC patients treated at a single center in an adjuvant setting, while previous studies focused mostly on a single adverse effect at a time [16,17] and included small series of patients often treated with additional agents to mitotane [18,19]. Therefore, we were able to provide an estimate of the frequency and time of onset of the different adverse effects, and we also monitored the changes induced by the introduction of specific therapies. We selected patients treated in an adjuvant setting, while being free of disease; so our findings were not influenced by the confounding effects of possible tumor hormonal secretion, cancer wasting syndrome or concomitant chemotherapy.

The topic of mitotane toxicity is of clinical interest considering that mitotane is still the backbone therapy for advanced ACC [15] and that the drug is also recommended as an adjuvant measure in ACC patients at high risk of recurrence following radical surgery [13,20,21,22,23,24]. Drug toxicity is a compelling issue particularly in the adjuvant setting, since it may be more difficult to justify treatment-related problems in patients who are free of disease. Prompt recognition and management of the unwanted effects of adjuvant mitotane therapy are key to make a long-term mitotane therapy feasible, improving a patient’s compliance and providing an acceptable quality of life.

Most of the unwanted effects are expected consequences of the drug mechanism of action, i.e., adrenal insufficiency due to the mitotane adrenolytic activity with inhibition of steroidogenesis [4] and hypercholesterolemia due to the stimulation of hydroxymethylglutarate-coenzyme A (HMGCoA) reductase [25,26]. However, a number of effects, such as alteration of thyroid and gonadal functions are less clearly understood. Literature data on the topic are limited and, to the best of our knowledge, evaluations of the outcome of specific therapies have not been undertaken yet. Despite the intrinsic limits due to its retrospective nature, the present study provides some interesting findings that expand previous knowledge.

Since it is held that mitotane causes cortisol deficiency, we did not focus on this specific effect that was the subject of a previous work of our group [27]. We observed, however, that fludrocortisone replacement was needed in only one third of mitotane-treated patients, confirming the view that the zona glomerulosa is more resistant to the adrenolytic action of mitotane [14]. Interestingly, after the introduction of fludrocortisone, PRA values were reduced in the majority of patients, while remaining high in some of them, as a likely hallmark of an insufficient replacement. This finding suggests that mitotane may enhance the metabolism of fludrocortisone, as is the case for glucocorticoids [28], thus requiring higher doses of both glucocorticoids and mineralocorticoids than in Addison disease.

We also confirmed that a deranged thyroid function is a rather frequent event, occurring in 36% of cases, being characterized by an early onset in most patients. In our cohort, only one patient received LT4 treatment due to subclinical hypothyroidism (TSH 6.5 uUI/mL and fT4 0.8 ng/dL). In all the remaining patients, LT4 therapy was started due to low levels of fT4 with low/normal TSH values—a pattern typical of central hypothyroidism as previously reported in literature [14,16]. The novel finding is that the introduction of levothyroxine is able to reduce TSH while causing a significative increase in fT4 levels. This signals an intact feedback mechanism and strengthens the concept that mitotane induces a central hypothyroid state [16,29].

The effect of mitotane on sexual function is gender specific, since 34% of male patients developed hypogonadism, while menstrual cycles were maintained in most women. The reason of this difference between genders is unclear. We can hypothesize a different effect of mitotane on the testicular isoenzyme 17β-hydroxysteroid dehydrogenase type 3, which is involved in the steroidogenesis of testicular Leydig cells, and the ovarian isoenzyme 17β-hydroxysteroid dehydrogenase type 1, which is involved in the steroidogenesis of theca and granulosa ovarian cells [30]. However, data about mitotane inhibition of these iso-enzymes are not currently available. It is important to underline that the measurement of total testosterone levels may fail to detect testosterone deficit due to the concomitant increase in SHBG, thus requiring assessment of free testosterone. Testosterone replacement was usually undertaken after more than 2 years of mitotane therapy. Since free testosterone levels dropped remarkably already in the first 6 months, it is likely that the delayed start of replacement of this hormonal deficit depends on a low priority value given by both physicians and patients to testosterone therapy. With the passing of time from a diagnosis of malignancy, the need of a more satisfactory sexual life becomes a more important issue. Moreover, testosterone deficit could contribute to asthenia and fatigue, which are commonly reported by mitotane-treated patients.

We found that up to 50% of women of childbearing age developed ovarian cysts upon treatment with mitotane, as previously reported in small case series [17,18]. It is of clinical importance to be aware of this unwanted effect of mitotane, because finding a growing ovarian mass induces anxiety and may conduce to a misdiagnosis of ACC progression. This finding supports the need to screen all women of childbearing age before starting mitotane therapy. Watchful waiting is enough in most cases, although ovarian cysts persist in mitotane therapy.

Half of patients developed dyslipidemia during mitotane therapy, in many cases after a few months from the start of treatment. Mitotane induces a peculiar lipid pattern characterized by a prominent increase in HDL rather than LDL cholesterol, and a concomitant increase in triglycerides, as previously reported [14,19]. It is unclear if this lipid profile will put mitotane-treated ACC patients at a higher risk of cardiovascular events. However, the protective role of HDL cholesterol has been recently questioned, since no interventional study has demonstrated that increasing HDL cholesterol is associated with a reduction in cardiovascular events [31]. This adds uncertainty to the choice of treating mitotane-induced dyslipidemia. In our cohort, the preferred lipid-lowering drug was rosuvastatin, because it is not metabolized through the CYP3A4 system, which is profoundly induced by mitotane [28]. We observed a significant difference between patients treated with rosuvastatin at the dose of 10 or 20 mg/daily. In the first group, the percentage reduction in LDL cholesterol was much lower than expected (−6% in our patients vs. −43% reported in the general population [32]), while in the second group, the reduction goal was achieved (−44% in our patients vs. −48% in the general population [32]). This new finding suggests that mitotane may interfere with the metabolism of rosuvastatin through CYP3A4 independent pathways.

After the introduction of supportive therapies, our patients generally reported an improvement in well-being and general conditions. However, we should disclose the limitation of not having performed a detailed assessment of patient-reported outcomes. As for other cancer types, it is time to move from an approach focalized only on preventing cancer progression towards a more comprehensive and patient-centered management. Such an approach should integrate patient-reported outcomes, which encompass data reported directly by patients about how they feel and function (i.e., symptoms, physical function and quality of life) for monitoring patient clinical status [33].

It is of note that a relevant number of patients developed side effects before the achievement of target mitotane levels (≥14 mg/dL). Therefore, this widely accepted cut-off for drug efficacy [11,12,13,34,35,36,37] should not be considered as a threshold for safety, and also patients exposed to lower mitotane levels should be careful monitored for unwanted events.

## 4. Materials and Methods

We conducted a retrospective analysis on 74 ACC patients followed at our center and treated with adjuvant mitotane after radical surgery from 2000 to 2018. To be included in the study, patients had to meet the following inclusion criteria: age 18 years or older at the time of diagnosis; histologically confirmed diagnosis of ACC (based on Weiss score [38]); ENSAT stage I-III ACC [39]; complete tumor resection, defined on the basis of surgical report, pathology report and postoperative imaging; treatment with mitotane for at least 12 months. Exclusion criteria were: mitotane therapy before surgery; discontinuation of mitotane therapy for more than 2 weeks for any reason during the study period; use of other medications interfering with mitotane effect (spironolactone, ketoconazole, tyrosine kinase inhibitors [40]); or chemotherapy. Patients with recurrent disease were censored at the time of ACC relapse if they were treated with other systemic therapy (e.g., chemotherapy), while patients treated with loco-regional therapy (e.g., radiofrequency) were not censored assuming that this type of treatment would not interfere with the biochemical effects of mitotane.

Follow up was interrupted at the time of mitotane discontinuation, due to whatever reason, or at the time of tumor progression if this led to the introduction of a systemic therapy different from mitotane. Follow up for this study was closed in July 2018.

In our practice, we use a low-dose starting mitotane regimen, because it seems better tolerated. We start with a dose of 1 g daily with further daily increments of 0.5 g (1 tablet) every 4 days until the maximal tolerated dose—usually of 6 g or less—targeting serum mitotane concentrations of 14–20 mg/L is reached. A general measure to deal with mitotane toxicity is a step down to the previously tolerated dose, or temporary drug withdrawal in the event of severe manifestations. In case of serum mitotane levels exceeding the target range, we reduce the dose according to peak levels and accompanying symptomatology [41,42].

At our center, treated patients adjuvantly undergo a first visit at 1 month from the start of mitotane (for laboratory work-up and clinical evaluation), then a second visit 2 months later and the following visits every 3 months during the first 2 years of treatment, with imaging, laboratory work-up, endocrine assessment (see below) and clinical evaluation. Additional visits are performed if clinically indicated. Moreover, we keep in close contact with patients, using emails and phone calls, to support them and provide information on how to deal with the eventual adverse events of therapy.

Laboratory work-up includes blood cell count, hemoglobin, electrolytes, liver and kidney function tests, lipids and mitotane plasma levels.

All data were obtained by reviewing patient history, medical records and source documents. Since the study was conducted at the San Luigi Gonzaga Hospital—University of Turin, an academic center where observational retrospective studies do not require the specifical approval of the ethical committee, the copy of the ethical approval is not available. We propose to any patient with ACC followed up in our center a general informed consent, and all patients included in this study signed it.

Data were processed by skilled and experienced personnel using a specifically tailored data form. We collected data on clinical and demographical characteristics, date and type of surgery, stage at diagnosis, pathology reports, date of start and stop of mitotane treatment. The following endocrine tests were evaluated for the purpose of the study: TSH, fT4, PRA, aldosterone, serum cortisol, ACTH, testosterone, SHBG, FSH, LH, at baseline (before introducing mitotane) and then after 6, 12 and 24 months of therapy. Total cholesterol, HDL cholesterol and triglycerides were also recorded at the same times. We also registered the date of the start of any replacement therapy and its type (hydrocortisone, fludrocortisone, levothyroxine, or testosterone in men), and the date of the start of lipid lowering therapy. We assumed mineralocorticoid deficiency in the presence of one or more of the following conditions: (i) elevated PRA with low/normal aldosterone levels; (ii) unexplained hyperkalemia or hyponatremia, and both; (iii) (orthostatic) arterial hypotension. In fertile women, we registered any menstrual irregularity and occurrence of ovarian cysts.

We evaluated how the following biochemical variables were modified during the first two years of mitotane therapy, because this is the duration of treatment recommended for adjuvant therapy [15,43]. At our center, however, mitotane therapy is continued even longer, if tolerated. For any variable of interest, we considered, separately, the period of time during which patients did not undergo a specific (replacement) therapy form that after institution of treatment. In such case, we evaluated whether any change in the biochemical variables occurred after 6 months of specific treatment.

All biochemical analyses were performed at the San Luigi Gonzaga Hospital using commercial reagents. Serum cortisol was determined using chemo-luminescence (Immunolite 2000, DPC, Los Angeles, CA, USA); plasma ACTH was determined using IRMA (Scantlbodies Laboratory Inc., Santee, CA, USA); PRA and aldosterone were measured with RIA (Sorin Biomedica, Saluggia, Italy and Adlatis, Bologna, Italy, respectively); testosterone, FSH, LH, TSH and fT4 were measured using an automated chemo-luminescence system (Abbott Architect ci 8200). Mitotane concentrations were retrieved from the Lysosafe Online^®^ database, available at www.lysosafe.com [13].

All statistical analyses were performed by using the “Statistica” software of the Windows package (Statsoft Inc., Tulsa, OK, USA). Rates and proportions were calculated for categorical data, and the median and ranges for continuous data. Friedman ANOVA was used to compare biochemical values during mitotane therapy; the Wilcoxon test was used to compare hormone values before and after six months of replacement therapy. All reported *p*-Values are two-sided. *p*-values of less than 0.05 were considered as statistically significant.

## 5. Conclusions

In conclusion, our study underlines the complexity of the management of adjuvant mitotane therapy, which requires the institution of multiple replacement therapies to deal with different adverse effects associated with treatment. The observation that the adverse effects may occur frequently before the achievement of target mitotane concentrations underlines the need of a close clinical and biochemical monitoring also in patients with low mitotane levels. A careful and global approach is key to take care of mitotane toxicity, making mitotane therapy safer and easier for patients. The study shows that the replacement therapies can revert biochemical alterations induced by mitotane and calls for a prospective evaluation of patient-reported outcomes during adjuvant mitotane treatment.

## Figures and Tables

**Table 1:** Characteristics of patients.

Characteristics	Valid Cases (*N*)	Values
Sex, N (%)	74	
Male		35 (47.3%)
Female		39 (52.7%)
Age at diagnosis, years	74	
Median (range)		46 (18–77)
Tumor stage at diagnosis, N (%)	74	
Stage I		10 (13.5%)
Stage II		56 (75.7%)
Stage III		8 (10.8%)
Hormone secretion at diagnosis, N (%)	74	
No		36 (48.6%)
Yes		38 (51.4%)
Glucocorticoid		21 (55.3%)
Androgen		8 (21.0%)
Glucocorticoid + Androgen		6 (15.8%)
Other		3 (7.9%)
Ki67 at diagnosis	67	
Median (range)		20 (5-70)
≤10%		17 (25.4%)
>10%		50 (74.6%)
Weiss at diagnosis	67	
Median (range)		6 (3–9)
Duration of mitotane therapy, months	74	
Median (range)		40 (12–195)

**Table 2:** Supportive therapies.

Treatment	Treated Patients (%)	Months from Mitotane Start Median (Range)
Hydrocortisone/Cortisone acetate	100	0 (0–0)
Fludrocortisone	32.4	10 (0–119)
Levothyroxine	36.2	9 (2–71)
Testosterone (men)	34.3	33 (5–78)
Lipid-lowering therapy	50.0	6 (0–57)

**Table 3:** Disturbance of gonadal function in fertile women.

N 26 Patients	N (%)
Ovarian cysts	20 (76.9)
Known before mitotane start	3 (11.5)
New onset during mitotane therapy	17 (65.4)
Treatment of ovarian cysts	
Follow-up	14 (73.7)
Surgery	2 (10.5)
Transcutaneous drainage	1 (5.3)
Medical therapy (EP)	2 (10.5)
Menstrual irregularities	8 (30.8)
Spotting	4 (15.4)
Metrorrhagia	2 (7.7)
Oligomenorrhea	2 (7.7)
Treatment of menstrual irregularities	
Medical therapy (EP)	5 (62.5)
Follow-up	3 (37.5)

EP = Estrogen–Progesterone.

**Table 4:** Time relationship between the onset of side effects and the achievement of target mitotane levels.

Side Effects	Valid Cases	Months from First Mitotane levels ≥ 14 g/dL Median (Range)	Patients Developing Side Effects before Achievement of Mitotane Levels ≥ 14 g/dL N (%)
Mineralocorticoid deficit	19/24	3 (−24–114)	6 (31.6)
Hypothyroidism	25/25	3 (−63–65)	9 (36)
Male hypogonadism	12/12	6 (1–52)	0 (0)
Dyslipidemia	32/35	0 (−65–54)	16 (50)
Ovarian cysts	17/17	3 (−10–81)	5 (29.4)
